# Porcine Hemagglutinating Encephalomyelitis Virus Triggers Neural Autophagy Independently of ULK1

**DOI:** 10.1128/JVI.00851-21

**Published:** 2021-09-09

**Authors:** Zi Li, Feng Gao, Yungang Lan, Jiyu Guan, Jing Zhang, Huijun Lu, Kui Zhao, Wenqi He

**Affiliations:** a Key Laboratory of Zoonosis Research, Ministry of Education, College of Veterinary Medicine, Jilin University, Changchun, China; b Key Laboratory of Zoonosis Research, Ministry of Education, Institute of Zoonosis, Jilin University, Changchun, China; Loyola University Chicago

**Keywords:** coronavirus, porcine hemagglutinating encephalomyelitis virus, autophagy, ULK1, autophagosome, BECN1, ULK1

## Abstract

Uncoordinated 51-like kinase 1 (ULK1) is a well-characterized initiator of canonical autophagy under basal or pathological conditions. Porcine hemagglutinating encephalomyelitis virus (PHEV), a neurotropic betacoronavirus (β-CoV), impairs ULK1 kinase but hijacks autophagy to facilitate viral proliferation. However, the machinery of PHEV-induced autophagy initiation upon ULK1 kinase deficiency remains unclear. Here, the time course of PHEV infection showed a significant accumulation of autophagosomes (APs) in nerve cells *in vivo* and *in vitro*. Utilizing ULK1-knockout neuroblastoma cells, we have identified that ULK1 is not essential for productive AP formation induced by PHEV. *In vitro* phosphorylation studies discovered that mTORC1-regulated ULK1 activation stalls during PHEV infection, whereas AP biogenesis was controlled by AMPK-driven BECN1 phosphorylation. A lack of BECN1 is sufficient to block LC3 lipidation and disrupt recruitment of the LC3-ATG14 complex. Moreover, BECN1 acts as a bona fide substrate for ULK1-independent neural autophagy, and ectopic expression of BECN1 somewhat enhances PHEV replication. These findings highlight a novel machinery of noncanonical autophagy independent of ULK1 that bypasses the conserved initiation circuit of AMPK-mTORC1-ULK1, providing new insights into the interplay between neurotropic β-CoV and the host.

**IMPORTANCE** The ongoing coronavirus disease 2019 (COVID-19) pandemic alongside the outbreaks of severe acute respiratory syndrome (SARS) and Middle East respiratory syndrome (MERS) pose *Betacoronavirus* (β-CoV) as a global public health challenge. Coronaviruses subvert, hijack, or utilize autophagy to promote proliferation, and thus, exploring the cross talk between β-CoV and autophagy is of great significance in confronting future β-CoV outbreaks. Porcine hemagglutinating encephalomyelitis virus (PHEV) is a highly neurotropic β-CoV that invades the central nervous system (CNS) in pigs, but understanding of the pathogenesis for PHEV-induced neurological dysfunction is yet limited. Here, we discovered a novel regulatory principle of neural autophagy initiation during PHEV infection, where productive autophagosome (AP) biogenesis bypasses the multifaceted regulation of ULK1 kinase. The PHEV-triggered noncanonical autophagy underscores the complex interactions of virus and host and will help in the development of therapeutic strategies targeting noncanonical autophagy to treat β-CoV disease.

## INTRODUCTION

Autophagy is an adaptive response to environmental stresses and a survival mechanism involving initiation, cargo recruitment, autophagosome (AP) maturation, and AP-lysosome/endosome fusion. Autophagy is routinely described in two ways: canonical autophagy and noncanonical autophagy (1). The first occurs via the mechanistic target of the receptor tyrosine kinase (RTK)/phosphatidylinositol 3-kinase (PI3K)/AKT/mechanistic target of rapamycin complex 1 (mTORC1) signaling pathway (2). During canonical autophagy, AP formation requires the action of a conserved set of factors that act in a hierarchical manner. These factors are also referred to as core machinery, including the ULK1 kinase complex, PI3K complex 1 (PI3Kc1) containing Beclin-1 (BECN1)/ATG14 subunit, ATG2, WD repeat proteins interacting with phosphoinositides (WIPIs), and ATG8-ATG12 conjugation systems (3). ULK1/Atg1 clustering at the site of AP formation initiates canonical autophagy, and its activation is regulated by the cellular nutrient sensor kinases AMPK and mTORC1 in a feedback manner (4, 5). AMPK and the core autophagy machinery are linked once AMPK directly phosphorylates ULK1, consequently governing the progression of AP formation that captures specific cargo material (6).

Noncanonical autophagy is triggered by mTOR-independent signals such as TRP ion channel mucolipin (TRPML), c-Jun N-terminal kinase (JNK), reactive oxygen species (ROS), and microRNAs (2, 7–9). It can occur under pathological circumstances (e.g., pathogen invasion) and is carried out through various complex mechanisms because AP biogenesis does not require the hierarchical activity of all ATG proteins or lipid kinases (9, 10). For example, hepatitis B virus X protein (HBx) promotes the generation of ROS to induce noncanonical AP formation, indicating that the upstream mTOR-ULK1 signaling axis is not the only route by which bona fide autophagy is initiated (10). Growing evidence indicates that the canonical and noncanonical autophagy pathways are variations in the common theme of self-eating. That is, autophagy induction and the components required vary in terms of the use of certain ATG proteins and membrane structures employed, although some alternative mechanisms remain under debate.

Outbreaks of severe acute respiratory syndrome (SARS), Middle East respiratory syndrome (MERS), and coronavirus disease 2019 (COVID-19) highlight the significance of understanding betacoronaviruses (β-CoVs) as worldwide health challenges. β-CoVs, including SARS-CoV-2, SARS-CoV, MERS-CoV, porcine hemagglutinating encephalomyelitis virus (PHEV), and murine hepatitis virus (MHV), usually cause respiratory or gastrointestinal illness and even neurologic manifestations. Currently, extensive advances have been made in discovering the intricate network that controls autophagy triggered by β-CoVs (11–14). Fortunately, great efforts have been made by the research community to discover therapeutic antiviral strategies for coping with such emergencies, including the controversial drug chloroquine, which targets the autophagy process (15–17). MHV, a known neurotropic β-CoV, sequesters APs that gather two endoplasmic reticulum (ER)-associated degradation (ERAD) regulators into its replication and transcription complexes, thereby exploiting autophagy for viral replication (18). Another highly neurotropic β-CoV, PHEV, often works as a safe model virus to study the mechanism of coronavirus infection and neuronal injury (19, 20). PHEV infects mainly suckling pigs, causing encephalomyelitis and/or vomiting and wasting disease (VWD) with a mortality rate of 30% to 100% (21, 22). The virus propagates in nerve cells of the host central nervous system (CNS), resulting in acute neurodegeneration in young rats (23). A recent study uncovered that PHEV could induce noncanonical autophagy with lysosomal dysfunction, thereby facilitating viral pathogenicity (24). However, how PHEV triggers the initial signal and subsequently dictates autophagy-specific intracellular events is far from understood. Moreover, vast clinical setbacks urge a better understanding of viral subversion of autophagy.

Here, using PHEV as a model β-CoV, we focus on the noncanonical machinery of neural AP formation during virus infection. By generating ULK1-knockout (ULK1-KO) N2a cells, we demonstrated that the AMPK-mTORC1-ULK1 axis, which is essential in the canonical autophagy pathway, is not necessary for autophagy initiation induced by PHEV. Instead, the virus employs AMPK-driven BECN1 phosphorylation as a node to integrate incoming autophagy signals for AP biogenesis, bypassing the ULK1 activation step. Our findings describe a novel mechanism of ULK1-independent neural autophagy that interacts with neurotropic β-CoV, contributing to the understanding of the pathogenesis of coronavirus-induced nerve injury.

## RESULTS

### PHEV induces irregular DMV accumulation in nerve cells of mice.

**FIG 1:**
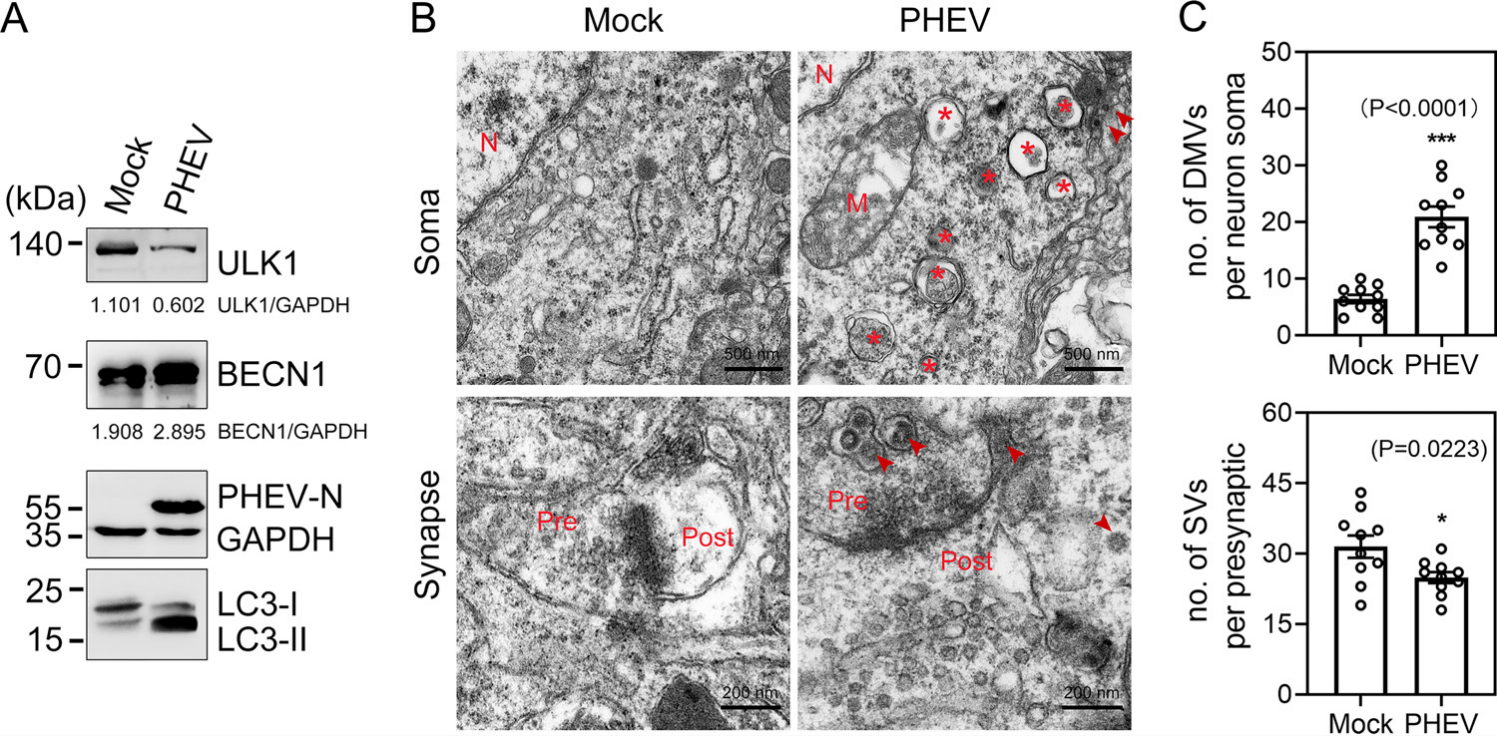
DMVs appear in the CNS of mice during PHEV infection. (A) Western blot analysis confirmed that the expression of both LC3-II and BECN1 was increased in hippocampal brain lysates from PHEV-infected mice compared to mock-infected mice. (B) TEM analysis of somas and synapses of hippocampal neurons from PHEV-infected versus mock-infected mice. Representative micrographs are shown with the indicated scale bars (500 nm, 200 nm). Virions are marked by arrowheads. Asterisks indicate DMVs. M, mitochondria; N, nuclei; Pre, presynaptic element; Post, postsynaptic element. (C) The number of DMVs and SVs was quantified using ImageJ (*n* = 5 technical replicates). Means ± standard deviations (SD) are shown. Unpaired two-tailed Student's *t* test; *, *P* < 0.05; ***, *P* < 0.001.

AP biogenesis involves *de novo* formation of a membrane that elongates to sequester cytoplasmic cargo and closes to form a double-membrane vesicle (DMV). Since the benefit of APs was observed during PHEV infection *in vitro* (24), we investigated whether the virus altered membrane rearrangement in the CNS of mice. Three-week-old BALB/c mice were infected with 100 μl PHEV (10^4.45^ 50% tissue culture infective doses [TCID_50_]/0.1 ml). The mice died within a week of infection and exhibited typical neurological dysfunction symptoms starting at approximately 3 days postinfection (dpi). Treatment with PHEV significantly enhanced the amount of lipidated LC3-II and BECN1 in the hippocampal neurons of mice (Fig. 1A). At 5 dpi, the ultrastructure of membrane modifications in the mouse brain was visualized by transmission electron microscopy (TEM). For PHEV-infected mice, the cytoplasm of hippocampal neurons displayed overt accumulation of DMVs (having morphological resemblance to APs) encircling amorphous structures, which appeared to be assembled virions, undigested organelles, or degraded debris (Fig. 1B and C). Ultrastructural alterations were also found in synapses, including decreased numbers of synaptic vesicles and narrowed synaptic clefts, likely due to impaired membrane trafficking from the soma (Fig. 1B and C). The number of synaptic vesicles in the presynaptic portion was quantified by ImageJ (Fig. 1C). The cellular architecture and organelle appearance were intact in mock-infected mice (Fig. 1B). These data suggested that PHEV infection induces AP-like DMV formation in the nerve cells of mice under physiological conditions.

### Loss of ULK1 kinase does not impair productive AP formation in nerve cells.

**FIG 2:**
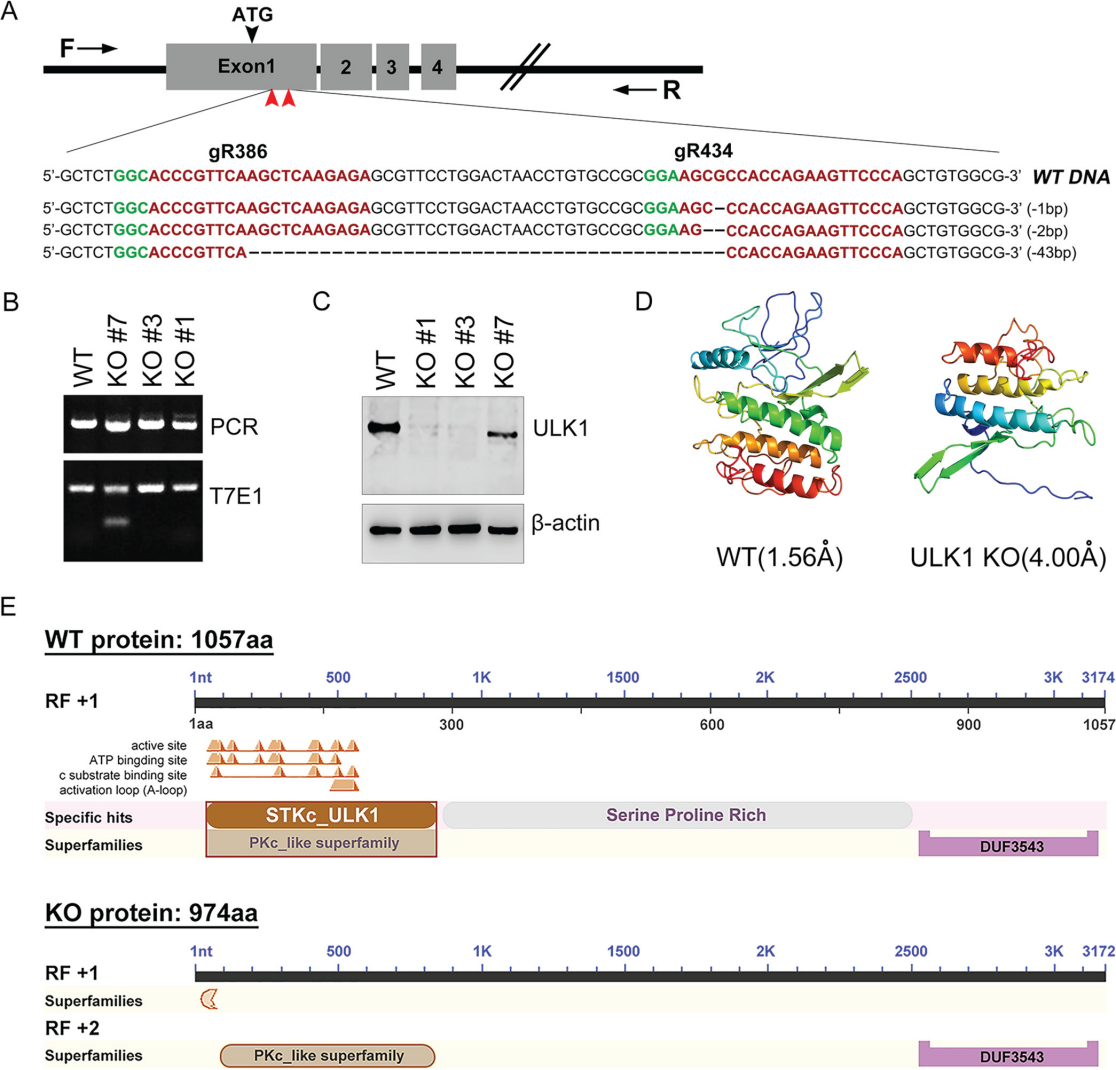
Generation and identification of ULK1-KO N2a cells. (A) Targeted strategy to generate a loss-of-function ULK1 clone using CRISPR/Cas9 gene targeting. Two sgRNAs (gR386 and gR434) targeting sequences in exon 1 of ULK1 (red arrowheads) were designed. Protospacer adjacent motif (PAM) sequences are indicated in green, and small guide RNA (sgRNA) sequences are indicated in red. (B) Genomic DNA was extracted from ULK1-KO N2a cells and amplified by PCR using the genotyping primers (black arrows) shown in panel A. Amplicons from colonies KO#1, KO#3, and KO#7 were subjected to the T7E1 assay and Sanger sequencing. (C) Western blot assay of the amounts of ULK1 protein from selected WT and KO clones. (D) Cartoon representation of the crystal structure of the conserved domain in WT and mutant ULK1. The image is colored by rainbow from the N to the C terminus. WT (1.56 Å) model dimensions (in angstroms): X, 62.875; Y, 57.652; Z, 39.296. Mutant (4.00 Å) model dimensions (in angstroms): X, 61.345; Y, 55.875; Z, 43.159. (E) Encoded proteins and conserved domains in WT ULK1 and CRISPR/Cas9-edited ULK1 were predicted. aa, amino acids.

The ULK1 kinase complex is one of the most upstream-acting components of the autophagy machinery and is usually activated to initiate the formation of APs, which capture cargo material (3, 25). To analyze the role of canonical autophagy signaling and its regulation by ULK1 in a genetically defined background, we first generated a ULK1-KO N2a cell line by using the CRISPR/Cas9 gene editing technique (Fig. 2A). Two of 12 independent homozygous ULK1-KO clones (KO#1 and KO#3) lost both wild-type (WT) alleles, and one colony (KO#7) carried a heterozygous allele (Fig. 2B). Endogenous ULK1 protein expression deficiency was subsequently confirmed, and no significant difference in knockout efficiency between the KO#1 and KO#3 clones was observed (Fig. 2C). Analysis of the conserved domains and three-dimensional (3D) model dimensions (Å) predicted that the mutant clone (KO#3) exhibits a strong loss of function, as exon 1 deletion introduced a frameshift that eliminated the catalytic domain of the serine/threonine kinase ULK1, leading to the loss of kinase and autophosphorylation activity (Fig. 2D and E). Thus, we demonstrated efficient silencing of the endogenous ULK1 gene targeted at the DNA level by the CRISPR/Cas9 system in N2a cells.

**FIG 3:**
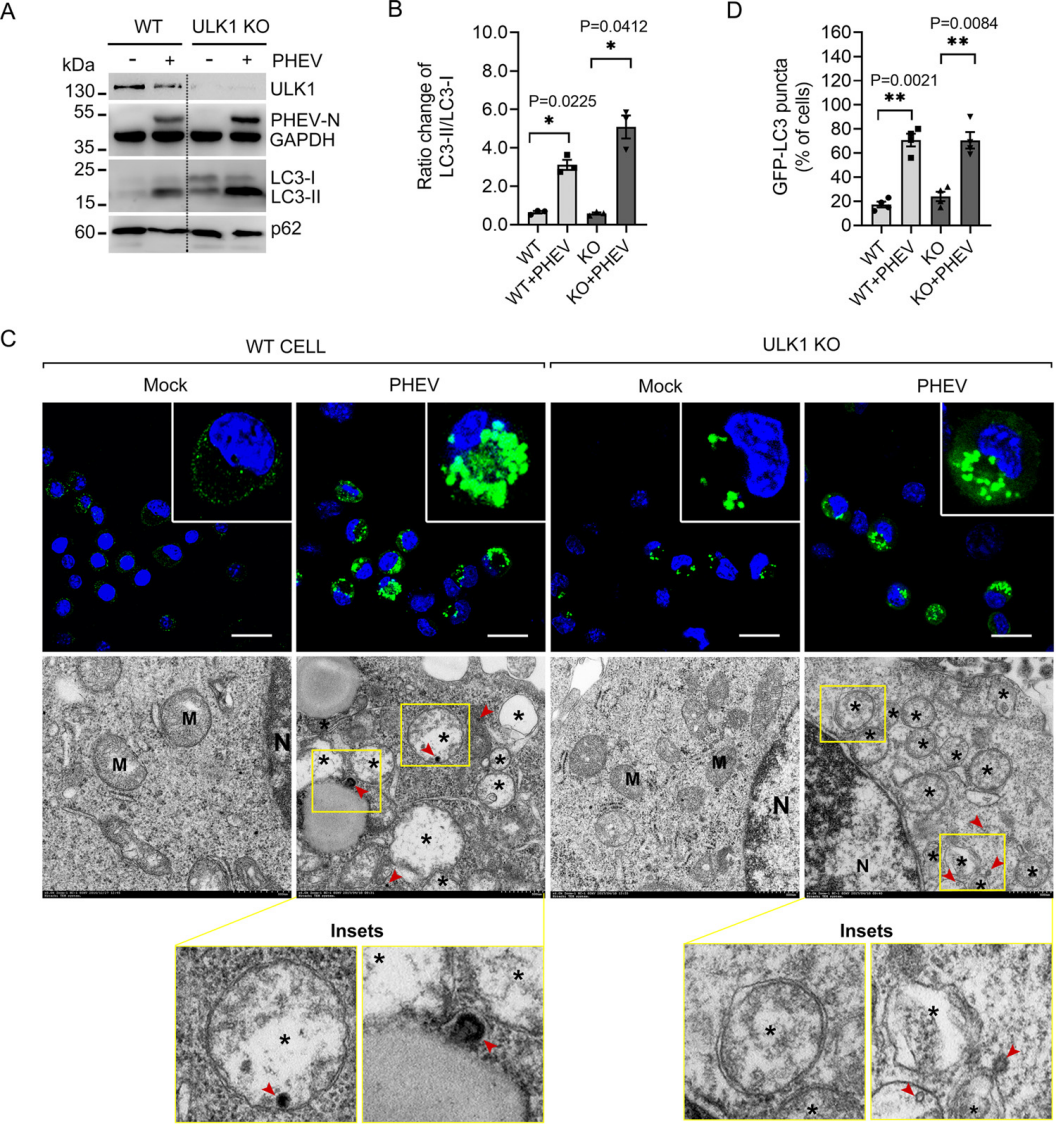
Knockout of ULK1 does not impair AP biogenesis. (A) WT and ULK1-KO (KO#3) colonies were incubated with or without PHEV for 48 h, and the cell lysates were detected by Western blot analysis. (B) LC3-II/LC3-I ratios were quantified by ImageJ and are shown in a histogram (*n* = 3; *, *P* < 0.05; one-way ANOVA). (C) GFP-LC3-expressing WT and ULK1-KO N2a cells were subjected to PHEV infection for 48 h. APs were measured by confocal laser scanning microscopy. Scale bars, 50 μm. Cells that were treated as indicated above were fixed and analyzed by TEM. Asterisks indicate single- and double-membrane vesicles, and PHEVs are indicated by red arrowheads. M, mitochondria; N, nuclei. Scale bars, 500 nm. (D) The percentages of cells containing >15 GFP-LC3 puncta were counted and are shown in a histogram (*n* = 4; **, *P* < 0.01; one-way ANOVA).

PHEV is sufficient to impair ULK1 kinase (26), so we asked whether the AP biogenesis occurs independently of ULK1. WT and ULK1-KO N2a cells were infected with PHEV at a multiplicity of infection (MOI) of 50. At 48 h postinfection (hpi), PHEV caused the conversion of cytosolic LC3-I in WT N2a cells to lipid-bound LC3-II, which also occurred in ULK1-KO N2a cells (Fig. 3A and B). Next, we tested the redistribution of ectopically expressed green fluorescent protein (GFP)-tagged LC3, and representative micrographs show that PHEV infection increased the number of GFP-LC3-positive APs in WT and ULK1-KO N2a cells (Fig. 3C and D). By investigating the presence of APs by transmission electron microscopy (TEM), we observed membrane rearrangement and accumulation of AP-like vesicles in both WT and ULK1-KO N2a cells after PHEV infection (Fig. 3C, lower panels). The findings suggest that PHEV-induced productive AP formation in nerve cells is independent of ULK1 kinase expression.

### PHEV-induced AP formation occurs independently of ULK1 phosphorylation.

**FIG 4:**
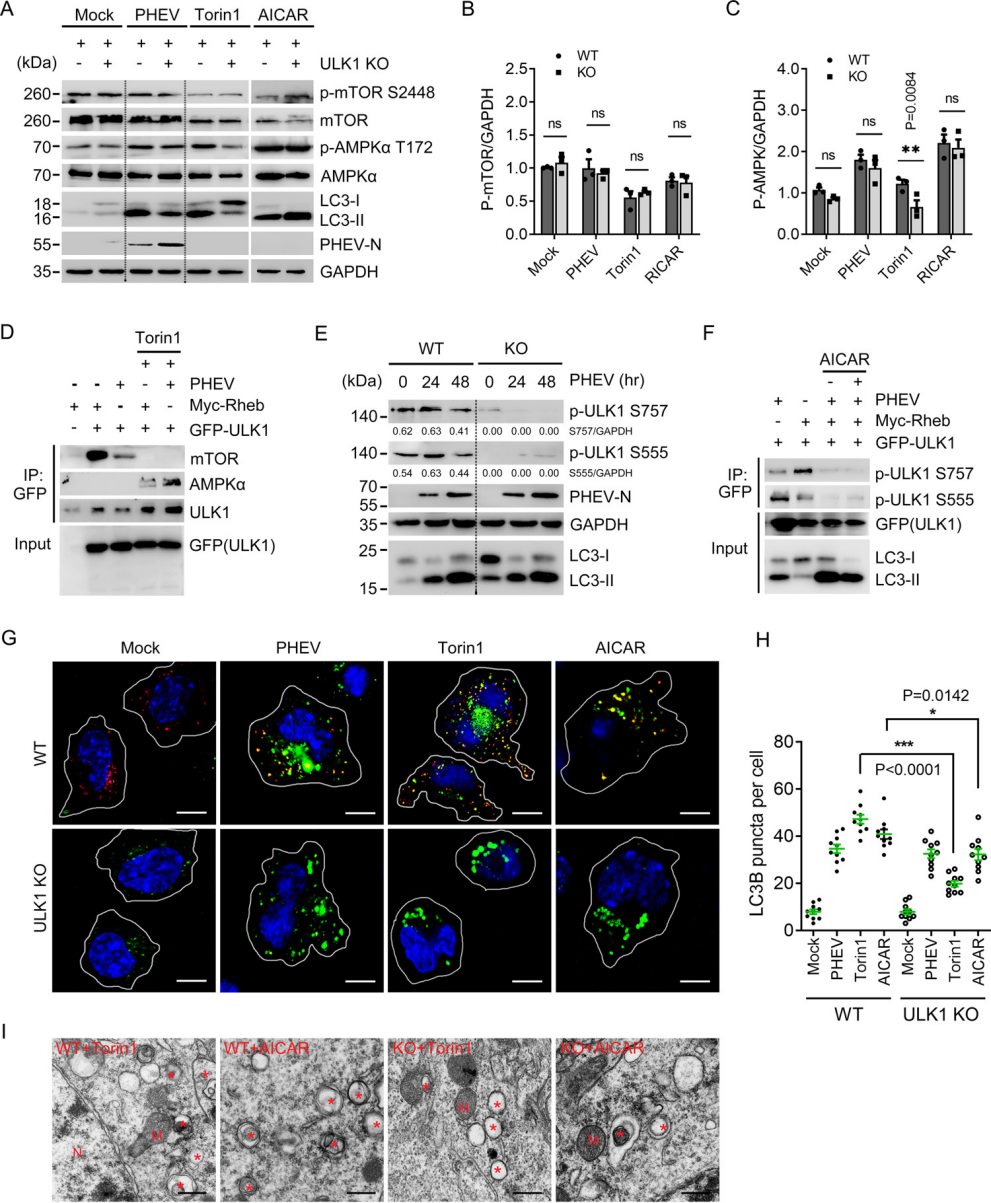
Recruitment and phosphorylation of ULK1 are not required for PHEV-induced autophagy. (A) WT and ULK1-KO N2a cells were subjected to PHEV infection for 48 h or treated with 50 nM Torin1 or 2 mM AICAR for 6 h before lysis. Immunoblotting for p-mTOR S2448, mTOR, p-AMPK T172, total AMPK, LC3-I/II, PHEV-N, and GAPDH was performed. (B and C) The ratio of p-mTOR or p-AMPK to GAPDH band intensities was analyzed using ImageJ (*n* = 3 technical replicates). (D) GFP-tagged ULK1 and Myc-tagged Rheb were cotransfected into WT N2a cells as indicated. The cells were treated with 50 nM Torin1 for 6 h or infected with PHEV for 48 h before lysis. GFP-ULK1 was immunoprecipitated, and the co-IP of AMPKα was determined by immunoblotting. (E) WT and ULK1-KO N2a cells were infected with PHEV for 0 to 48 h, and the phosphorylation of endogenous ULK1 at Ser757 and Ser555 was detected with MAbs against p-ULK1 S757 and p-ULK1 S555, respectively. (F) GFP-tagged ULK1 and Myc-tagged Rheb were cotransfected into WT N2a cells as indicated, followed by either AICAR or PHEV treatment. Total cell lysates were probed with antibodies against ULK1 phosphorylated at Ser555 or Ser757 and GFP. (G) Cells described in the legend for panel A were subjected to immunofluorescence analysis using antibodies against LC3B (green) and ULK1 (red). Nuclei were stained with DAPI (blue). Scale bars, 10 μm. (H) The total number of LC3B-positive puncta per cell was quantified using Harmony software on images acquired with an automated Operetta microscope. Three independent experiments were performed with approximately 10 cells counted per condition. Means ± SD are shown. Unpaired two-tailed Student's *t* test; *, *P* < 0.05; **, *P* < 0.01; ***, *P* < 0.001. (I) Cells that were treated with Torin1 or AICAR were detected by TEM. Asterisks indicate AP-like vesicles. M, mitochondria; N, nuclei. Scale bars, 500 nm.

Since the loss of ULK1 does not stall AP formation during PHEV infection, we asked whether the virus triggers autophagy independently of the AMPK-mTOR-ULK1 pathway. Activation of mTOR signaling was first monitored. As shown in Fig. 4A and B, Torin1, a potent and selective mTORC1/2 kinase inhibitor that activates mTOR-dependent autophagy, did not induce LC3 conversion in the absence of ULK1. Comparatively, global phosphorylation of mTOR (Ser2448) and LC3 lipidation were not inhibited in PHEV-infected ULK1-KO cells, suggesting that PHEV-induced neural autophagy does not depend on mTOR-ULK1 signaling (Fig. 4A and B). When analyzing the phosphorylation of AMPK, we found that PHEV increased the level of p-AMPKα (Thr172) irrespective of ULK1 deprivation (Fig. 4A and B). Treatment with 5-aminoimidazole-4-carboxamide ribonucleotide (AICAR), an AMPK-dependent autophagy activator, enhanced LC3 lipidation in ULK1-KO cells, as observed in PHEV-infected ULK1-KO cells (Fig. 4A and C). The data suggest that AMPK signaling is involved in PHEV-induced neural autophagy in a ULK1-independent manner.

Canonically, phosphorylation of ULK1 Ser555 by AMPK was identified to recruit phosphor-binding proteins, whereas active mTORC1 phosphorylates Ser757 to prevent the AMPK-mediated activation of ULK1 (6, 27, 28) To gain insight into the phosphorylation of ULK1 kinase within its activation loop, GFP-tagged ULK1 and a Myc-tagged Ras homologue enriched in the brain (Rheb) were cotransfected into N2a cells, followed by PHEV infection or Torin1 stimulation. We found that Rheb overexpression or PHEV infection suppressed the ULK1-AMPK interaction, which conversely increased after Torin1 stimulation (Fig. 4D). Since Rheb is an activator of the mTORC1 signaling pathway (29), our data suggest that the association ULK1-AMPK was strongly weakened by upstream mTORC1 activation during PHEV infection. To further understand the coordinated regulation of mTORC1- and AMPK-dependent PHEV infection, we tested the phosphorylation of endogenous ULK1 at Ser757 and Ser555. As shown in Fig. 4E, the absence of Ser757 and Ser555 phosphorylation did not stall LC3 lipidation in PHEV-infected ULK1-KO cells, indicating that mTOR- or AMPK-mediated ULK1 activation is not required for the progression of AP formation. These effects were more evident in coimmunoprecipitation (co-IP) assays conducted upon AICAR stimulation. We showed that ULK1 Ser555 phosphorylation and LC3 conversion were inhibited by Rheb overexpression, but autophagy was activated following PHEV infection (Fig. 4F). In addition, in the Rheb- and ULK1-overexpressing cells, AICAR treatment did not weaken the effect of PHEV infection (Fig. 4F). Therefore, our findings suggest that PHEV induces AP production but not through the canonical AMPK-mTORC1-ULK1 pathway.

ULK1 kinase acts not only to activate the autophagy machinery by phosphorylation but also to recruit it to the preautophagosomal structure/phagophore assembly site (PAS) for productive AP formation. After treatment with Torin1 or AICAR, the KO cells appeared to be almost absent in LC3-positive AP formation and ULK1 recruitment due to ULK1 deprivation (Fig. 4G and H). The ultrastructure of AP structures in the drug-treated cells was detected by TEM (Fig. 4I). In contrast, in PHEV-infected ULK1-KO cells, ULK1 rarely colocalized with LC3-positive APs following PHEV infection, although the number of APs was similar to that observed in PHEV-infected WT cells (Fig. 4G to I). This suggests that ULK1 recruitment is dispensable for PAS in PHEV-induced AP formation. Therefore, we conclude that PHEV-induced neural autophagy does not require the recruitment and activation of ULK1 at the cargo.

### Sensing of AMPK autophagic signals by BECN1 does not depend on ULK1.

**FIG 5:**
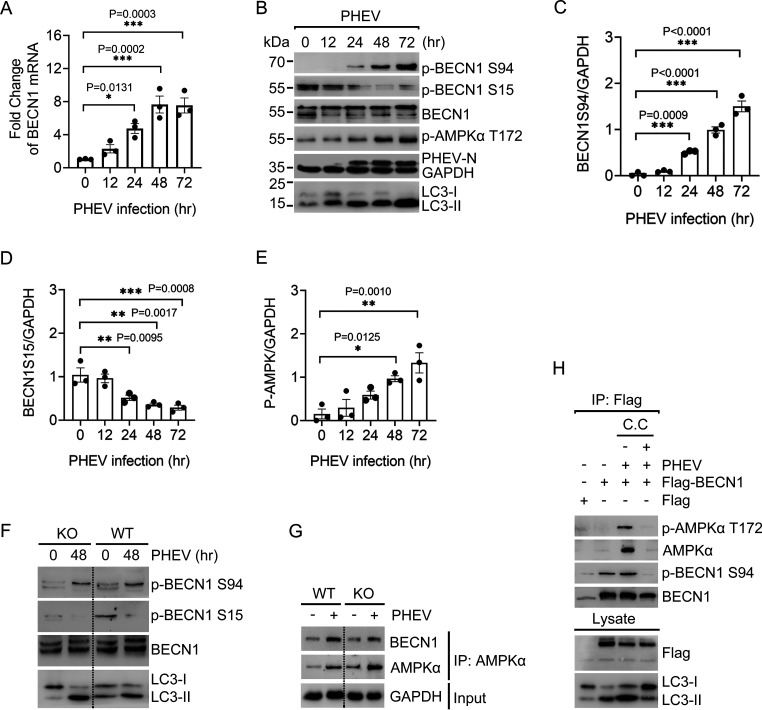
BECN1 is activated by AMPK in ULK1-independent autophagy. (A) Quantitative RT-PCR analysis of BECN1 mRNA levels in WT N2a cells after PHEV infection. (B) WT N2a cells were incubated with PHEV for the indicated duration. Western blot analysis was performed to monitor the total amounts of the indicated endogenous proteins and their modification. (C to E) Gray value analysis of the BECN1 Ser94 (C), BECN1 Ser15 (D), and P-AMPKα Thr172 (E) bands in panel B. (F) WT and ULK1-KO N2a cells were infected with PHEV for 0 or 48 h. Phosphorylation of endogenous BECN1 protein at Ser94 or Ser15 was determined by Western blot analysis. (G) AMPK-associated endogenous BECN1 protein was immunoprecipitated from WT or ULK1-KO N2a cell lysates following PHEV infection for 48 h. Immunoblotting was performed with the indicated antibodies. (H) WT N2a cells pretransfected with Flag-tagged BECN1 for 24 h were infected with PHEV (48 h), followed by treatment with C.C (10 μM, 6 h). Flag-BECN1 was affinity immunoprecipitated and immunoblotted with the indicated antibodies. Means ± SD are shown. Two-way ANOVA; *, *P* < 0.05; **, *P* < 0.01; ***, *P* < 0.001 (*n* = 3 technical replicates).

AP formation requires the action of a conserved set of hierarchical factors, of which BECN1 phosphorylation acts as a bridge between the ULK1 complex and the regulatory subunits relevant to autophagy triggering (30, 31). To explore the ULK1-independent core autophagy machinery, we investigated the significance of BECN1 phosphorylation in PHEV-induced neural autophagy. At the time points assayed, BECN1 mRNA production was strongly induced by PHEV (Fig. 5A). Western blot analysis showed that endogenous BECN1 was clearly phosphorylated at Ser94 following PHEV infection, correlating with AMPK activation and LC3 lipidation (Fig. 5B, C, and E). BECN1 Ser15 phosphorylation, in contrast, appeared to be downregulated (Fig. 5B and D). To gain insight into the lipid kinase activity of BECN1, we observed that ULK1 KO abolished the BECN1 phospho-Ser15 signal, while Ser94 phosphorylation was not significantly affected (Fig. 5F). These data demonstrate that BECN1 functions with redundancy in PHEV-induced neural autophagy upon ULK1 deprivation.

Given that PHEV infection activates AMPK (Fig. 4A), we hereafter tested whether BECN1 is a physiological target of AMPK and subsequently initiates autophagy. AMPK-associated BECN1 was immunopurified from WT and ULK1-KO N2a cells, and immunoblotting showed that the binding of AMPK with BECN1 was enhanced following PHEV infection (Fig. 5G). To rule out a correlation between BECN1 Ser94 phosphorylation and AMPK signaling to ULK1, the ULK1-KO N2a cells were pretransfected with Flag-tagged BECN1. Phosphorylation of BECN1 at Ser94 in the resultant immunoprecipitated complex was tested, and we found that PHEV induces the binding of p-AMPKα and p-BECN1 Ser94 in ULK1-KO cells (Fig. 5H). After BECN1 Ser94 phosphorylation was completely blocked by 10 μM compound C (C.C), an AMPK inhibitor, we observed inhibition of both LC3 lipidation and the AMPK-BECN1 interaction. The data showed that BECN1 acts as an important downstream substrate that senses AMPK signals in PHEV-induced autophagy. Collectively, these findings highlight that AMPK-driven BECN1 Ser94 phosphorylation is critical for integrating autophagy signals triggered by PHEV without the multifaceted regulation of ULK1.

### BECN1 phosphorylation acts as a driver of AP biogenesis.

**FIG 6:**
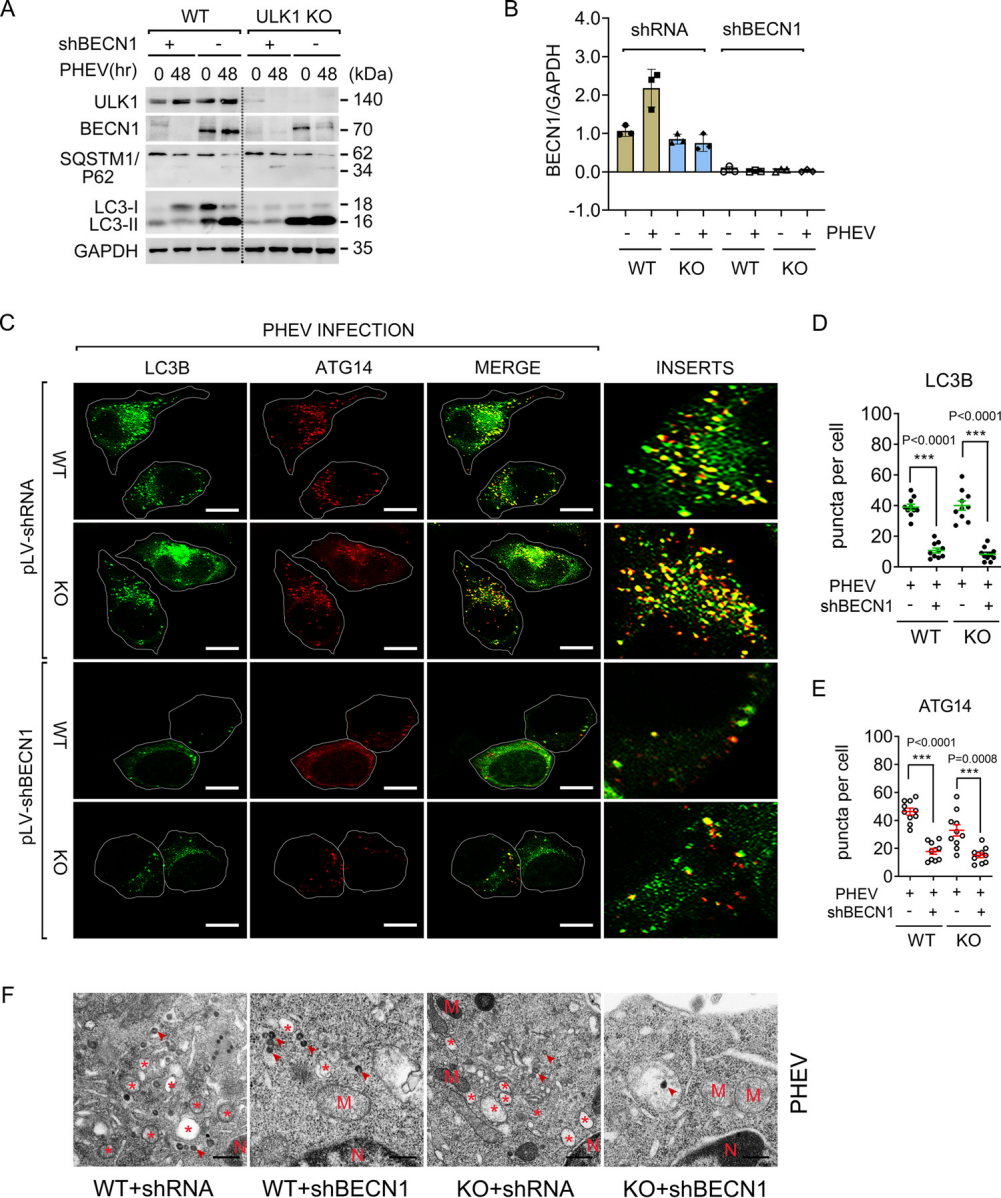
BECN1 is involved in PHEV-induced AP formation. (A) WT and ULK1-KO N2a cells were incubated with pLV-shBECN1 or pLV-shRNA and selected with puromycin for 1 week. Stably edited cells were then infected with PHEV for 48 h and assayed by Western blotting. (B) Quantitative analysis of the BECN1 bands in panel A using ImageJ (*n* = 3 technical replicates). (C) Immunostaining was performed in BECN1-silenced cells infected with PHEV for 48 h by using anti-LC3B (green) or ATG14 (red) MAb. Scale bars, 10 μm. (D and E) Quantitative analysis of LC3B- or ATG14-positive puncta per cell in panel C. (F) Stably edited cells that were treated as indicated in panel C were then assayed by TEM. Representative micrographs are shown. Virions are marked by arrowheads. Asterisks indicate AP-like vesicles. M, mitochondria; N, nuclei. Scale bars, 500 nm.

BECN1 is a component of PI3Kc1 and critically contributes to phagophore nucleation, which serves as a PAS (32–34). To elucidate the role of BECN1 in the ULK1-independent progression of AP formation, we employed an RNA interference (RNAi)-mediated knockdown approach to deplete BECN1. Cells transfected with pLV-shBECN1 efficiently blocked BECN1 expression and dramatically decreased LC3-II accumulation compared to that in the pLV-shRNA control group (Fig. 6A and B). By immunostaining for endogenous LC3, we found that WT and ULK1-KO cells harboring BECN1 knockdown exhibited a significant reduction in LC3-positive AP formation upon PHEV infection (Fig. 6C and D). To our knowledge, the BECN1-containing PI3Kc1 complex binds ATG14, and the subcomplex is localized to PAS, where it participates in AP formation (32). Upon scoring ATG14 localization, we observed a decrease in ATG14-positive APs during PHEV infection in BECN1-KO cells (Fig. 6C and E), indicating that the recruitment of BECN1-containing complexes to PAS is required for PHEV-induced AP formation. This effect was confirmed by a TEM assay (Fig. 6F).

**FIG 7:**
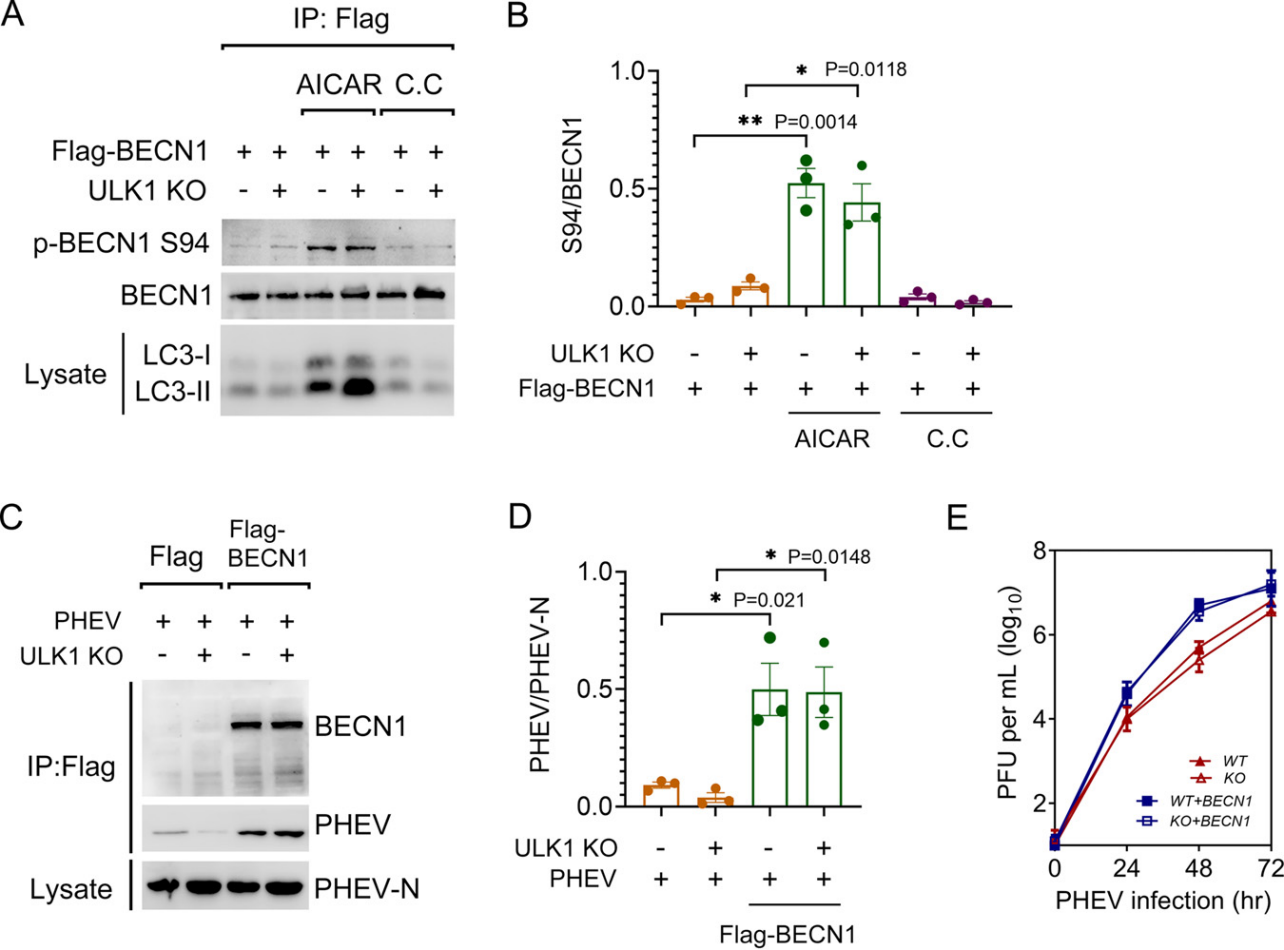
Ectopic BECN1 benefits PHEV replication. (A) WT and ULK1-KO N2a cells were transfected with Flag-BECN1 for 24 h, followed by treatment with 2 mM AICAR or 10 μM C.C. Cell lysates were subjected to the co-IP assay. (B) Quantitative analysis of BECN1 Ser94 from the immunoprecipitated proteins. (C) WT and ULK1-KO N2a cells expressing Flag-BECN1 were infected with PHEV for 48 h. Cell lysates were immunoprecipitated with anti-Flag IgG and assayed via immunoblotting with BECN1, PHEV-N MAbs, and PHEV polyclonal antibody. (D) Quantitative analysis of the immunoprecipitated PHEV. (E) Intracellular PHEV titers of cells treated as indicated for panel C were determined via plaque assay. Means ± SD are shown. Two-tailed unpaired Student's *t* test; *, *P* < 0.05; **, *P* < 0.01 (*n* = 3 technical replicates).

We next transiently expressed Flag-tagged BECN1 in WT and ULK1-KO N2a cells. As shown in Fig. 7A and B, AICAR stimulation significantly increased LC3-II accumulation but was decreased when BECN1 Ser94 phosphorylation was blocked following C.C treatment. This result suggested that BECN1 Ser94 phosphorylation could act as a driver of ULK1-independent AP biogenesis in nerve cells. Furthermore, ectopic expression of BECN1 increased both the viral titer and viral protein synthesis in comparison to those in the corresponding control cells (Fig. 7C to E). Together, these observations indicate that the BECN1-containing complex is involved in AP biogenesis independently of ULK1 during PHEV infection and that BECN1 may promote the process of PHEV replication through the autophagic pathway.

## DISCUSSION

**FIG 8:**
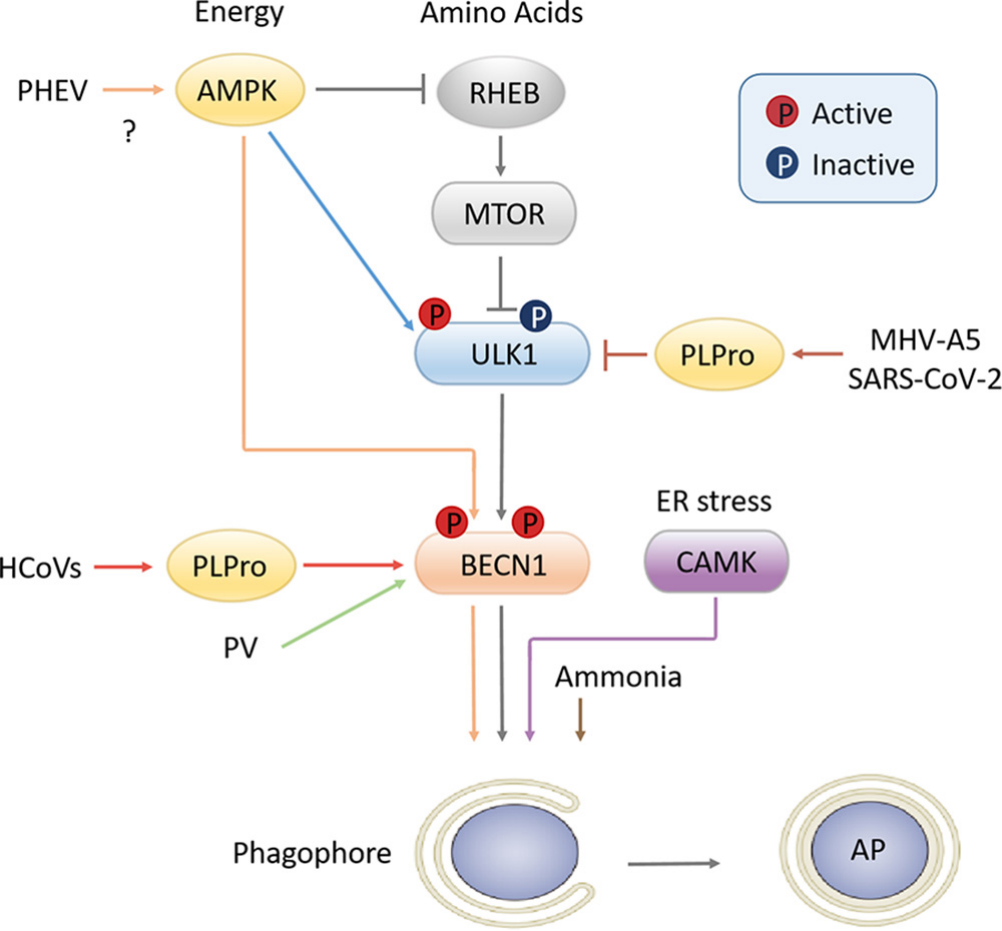
Schematic of autophagy initiation. The canonical autophagy pathway (gray arrows) in response to energy or amino acid signaling is depicted in the center. ULK1 kinase is negatively regulated by mTOR and phosphorylated by AMPK, subsequently activating the BECN1 complex and leading to AP formation. Comparatively, the noncanonical autophagy pathways vary. Papain-like protease (PLPro) of human coronavirus (HCoV) interacts with BECN1 to induce atypical autophagy (red arrows), while PLPro of MHV-A5 and SARS-CoV-2 cleaves ULK1 to disrupt host autophagy. Poliovirus (PV) induces autophagic signals bypassing the ULK1 complex (green arrow) in a BECN1-dependent manner. PHEV-induced neural autophagy requires AMPK-driven BECN1 signals bypassing mTOR inhibition or ULK1 phosphorylation, although the involved viral NSPs or genes are unclear (orange arrows). ER stress induces autophagy by activating CAMK1/4 (purple arrow), whereas the degradation process does not occur. Ammonia induces autophagy independently of ULK1, but the role of the BECN1 complex in this mechanism was not assessed (brown arrow).

The β-CoVs usually cause respiratory or gastrointestinal illness, and even neurologic manifestations, and are included on the World Health Organization (WHO) list of viruses most likely to cause future epidemics. PHEV is a neurotropic β-CoV and infects mainly suckling pigs, causing encephalomyelitis and/or vomiting and wasting disease (VWD) with a mortality rate of 30% to 100% (21, 22). The virus propagates in nerve cells of the host central nervous system (CNS), resulting in acute neurological dysfunction in young rats (23). Similar to MHV, PHEV is often used as a safe model β-CoV to study the mechanism of β-CoV infection and neuronal injury (18–20). In this paper, we provide new insights into and understanding of the molecular mechanisms of autophagy initiation and PHEV disease. From studies on ULK1-KO cells, we mapped a noncanonical autophagy initiation in response to PHEV infection (Fig. 8). That is, the biogenesis of APs does not always require ULK1 activation and mTORC1 suppression, during which a BECN1-containing complex acted as a driver. This is the first report of β-CoV-induced neural autophagy independent of ULK1 and might update the original definition of autophagy initiation.

Autophagy is a degradative process involved in nutrient and organelle turnover that starts with formation of the phagophore/omegasome (33). ULK1 is widely accepted as the primary upstream autophagy-specific kinase and functions in the initiation of autophagy (35). In the canonical autophagy pathway, ULK1 complex is negatively regulated by mTORC1 and is positively regulated by AMPK. However, significant loss of cellular ULK1 induced by neurotropic coronavirus has been described previously (26, 36). Therefore, how host autophagy responds to invading pathogens in the absence of canonical autophagy-regulating kinase is now of great interest. During PHEV infection, ULK1 expression that does not affect LC3 lipidation or AP formation was downregulated, suggesting that PHEV utilizes a novel autophagy initiation machinery independently of ULK1. The work of Velazquez et al. (37) also supports this conclusion, as they reported a defective autophagy signal that was independent of the ULK1 complex during poliovirus (PV) infection (Fig. 7). Most recently, Mohamud et al. (36) identified ULK1 as a novel bona fide substrate of SARS-CoV-2 papain-like protease (PLPro), which cleaves ULK1 to disrupt host autophagy. Since autophagy response is an integral part of the host immune system, it is therefore of little surprise that some viruses have evolved mechanisms to hijack or subvert the autophagy response.

AMPK, mTOR, and ULK1 are three interconnected major junctions within the autophagy signaling network. Cross talk, shortcuts, and feedback in the AMPK-mTOR-ULK1 pathway have been well established (6, 27). As a major nutrient/energy sensor, mTORC1-driven ULK1 phosphorylation physically weakens ULK1 activity under nutrient-rich conditions, but mTORC1 dissociates from the ULK1 complex upon starvation (38–40). Another nutrient-sensing regulatory kinase, AMPK, plays roles in autophagy induction by phosphorylating TSC2 and Raptor to inactivate mTOR or by directly interacting with ULK1 during glucose starvation (4, 6). At least 30 ULK1 phosphorylation events have been reported (e.g., at Ser555, Ser777, Ser637, Ser757, Thr574, Thr659, and Ser317), though few of them overlap (39, 41). In PHEV-induced neural autophagy, ULK1 Ser555 phosphorylation was not enhanced or counterbalanced by inhibitory Ser757 phosphorylation in nerve cells. These inconsistencies between our observations and the canonical autophagy pathway might reflect the alternative autophagic role of ULK1 upon the sensation of different triggers (i.e., glucose starvation versus virus invasion). Furthermore, our findings show a physical interaction between ULK1 and AMPK, indicating the initiation of both canonical (i.e., ULK1-dependent) and noncanonical (i.e., ULK1-independent) autophagy during PHEV infection. Such alternatives to the conserved scheme underscore the complex molecular aspects of neural autophagy and might provide additional opportunities for the virus to proliferate and survive in nerve cells.

BECN1 is the first mammalian autophagy protein identified as a novel Bcl-2-interacting protein, and its phosphorylation is required for PI3Kc1 (composed of VPS34, VPS15, BECN1, and ATG14) production (34). AMPK usually directly phosphorylates BECN1 or indirectly activates the BECN1 complex by interacting with ULK1 following amino acid withdrawal (30, 42, 43). Our work showed that BECN1 still acts as an important downstream substrate for sensing AMPK signals, following ULK1 deprivation. Since Ser15 has been identified as a phosphosite on BECN1 modified by ULK1 (31), its dephosphorylation in response to PHEV infection implies that some undefined targets have replaced ULK1-mediated BECN1 activation in this neural autophagy. It suggests that PHEV alters bioenergetic substrate (i.e., BECN1) to induce neural autophagy for its own survival when the function of canonical autophagy initiators (such as ULK1) is damaged.

Autophagy responses have varied effects on the outcome of viral infection. To date, knowledge of the autophagic signal network employed by coronavirus remains incomplete. It has been reported that the nonstructural protein (NSP) derived from several coronaviruses, such as NSP3 and NSP6, induces autophagy through direct binding to LC3 and BECN1 (44, 45). In comparison, PLPro of MHV-A5 and SARS-CoV-2 cleaves ULK1 to disrupt host autophagy (Fig. 7) (36). However, which NSPs are responsible for autophagy initiation over the duration of PHEV infection has yet to be investigated. Moreover, the intricate interplay between the regulation of BECN1-dependent and ULK1-independent pathways may also be a major obstacle for understanding neural autophagy. Thus, focusing on the development of therapeutic agents by targeting canonical/noncanonical autophagy processes will provide more insight into coronavirus-induced infectious disease control.

## MATERIALS AND METHODS

### Cells.

Mouse neuroblastoma cells (N2a, ATCC, CCL-131) were cultivated in high-glucose Dulbecco’s modified Eagle’s medium (DMEM) supplemented with 10% fetal bovine serum, 100 U/ml penicillin, and 100 μg/ml streptomycin. For transfection, cells were grown to 60% confluence and then transfected with the indicated plasmids using X-tremeGENE HP DNA transfection reagent (Roche, Sweden). For PHEV infection, virus stocks (strain HEV 67N; GenBank accession no. AY048917) were used in all experiments.

### Animals.

For the animal infection model, 3-week-old BALB/c mice (male) were intranasally inoculated with PHEV (10^4.45^ TCID_50_/0.1 ml). These mice were anesthetized with 3.5% chloral hydrate (1.0 ml/100 g), and their brain tissues were collected on ice. This study was approved by the Institutional Animal Care and Use Committee of the College of Veterinary Medicine, Jilin University, China (permission number KT201904002). All applicable institutional and/or national guidelines for the care and use of animals were followed.

### Western blot analysis.

Protein extracts were lysed in radioimmunoprecipitation assay (RIPA) buffer supplemented with 1 mM phenylmethylsulfonyl fluoride and phosphatase inhibitor cocktail 2, separated by SDS-PAGE, and then electrotransferred onto polyvinylidene difluoride membranes. Blots were blocked in 5% nonfat dry milk in Tris-buffered saline (TBS) (0.5 M, pH 7.4) with 0.2% Tween 20 for 1 h at room temperature and subsequently incubated with primary antibodies at 4°C overnight. The following primary antibodies were obtained from Cell Signaling Technology: Beclin-1 (1:1,000), phospho-Beclin-1 (Ser15) (1:1,000), phospho-Beclin-1 (Ser91/Ser94) (1:1,000), ULK1, phospho-ULK1 (Ser555), phospho-ULK1 (Ser757), AMPKα, phospho-AMPK (Thr172), mTOR, phospho-mTOR (Ser2448), LC3B, SQSTM1/p62, DYKDDDDK tag, MYC tag, and GFP tag. Antibodies recognizing GAPDH (glyceraldehyde-3-phosphate dehydrogenase) and beta-actin were kind gifts from Cell Signaling Technology. Mouse anti-PHEV-N monoclonal antibody (MAb) was provided by our research support group. Subsequently, blots were washed and probed with horseradish peroxidase (HRP)-conjugated secondary antibody (1:10,000) for 1 h at room temperature. The immunoreactive bands were visualized using ECL detection reagent (Millipore, Billerica, USA) quantified by ImageJ software.

### Co-IP assay.

For the co-IP assay, phosphate-buffered saline (PBS)-washed cell pellets were incubated with ice-cold 1× cell lysis buffer for 5 min, followed by sonication on ice three times for 5 s each. The lysates were then centrifuged for 10 min at 4°C and 14,000 × *g*, and the supernatants were collected. Then, 2.0 μg primary antibody was added to 200 μl cell lysate and incubated with gentle rocking overnight at 4°C. Twenty microliters of bovine serum albumin (BSA)-blocked Protein G Dynabeads was added to the lysate-antibody mix and incubated at 4°C for 3 h. The beads were washed five times with 1× cell lysis buffer, and protein-antibody complexes were eluted by boiling for 5 min at 95°C in 4× SDS sample buffer. Fifteen milligrams of cell lysates or 5 μl of immunoprecipitates was separated by SDS-PAGE, followed by Western blot analysis.

### qRT-PCR.

Total RNA was extracted, and reverse transcription was performed using PrimeScript reverse transcriptase (TaKaRa). Quantitative reverse transcription PCR (qRT-PCR) assays were conducted with SYBR Premix Ex Taq II (TaKaRa) on a CFX96 Touch real-time PCR detection system. The GAPDH gene was used as a housekeeping gene, and gene expression was calculated using the 2^−△△^*^CT^* method. PCR conditions were as follows: 95°C for 3 min, 95°C for 30 s, 60°C for 30 s, and 72°C for 30 s for a total of 45 cycles. Primers included GAPDH-F (5′-CTC AAC TAC ATG GTC TAC ATG TTC-3′), GAPDH-R (5′-ATT TGA TGT TAG TGG GGT CTC GCT C-3′), BECN1-F (5′-CAG CTC CAT TAC TTA CCA C-3′), and BECN1-R (5′-CCA TCA GAT GCC TCC CCG A-3′).

### Confocal fluorescence microscopy.

N2a cells that had been preplated in 12-well culture dishes were fixed in 4% paraformaldehyde for 10 min and permeabilized with 0.2% Triton X-100 for 10 min, followed by blocking with 5% nonfat dry milk for 1 h at room temperature. Primary antibodies, including ATG14 (1:500), LC3B (1:500), and ULK1 (1:500), were added and incubated overnight at 4°C. After washing with PBS, the cells were incubated with secondary antibodies, including mouse IgG conjugated to Alexa Fluor 488 and rabbit IgG conjugated to Alexa Fluor 594, for 1 h at room temperature. Nuclei were then labeled with 4′,6-diamidino-2-phenylindole (DAPI). All images were acquired randomly using a laser scanning confocal fluorescence microscope and quantitatively analyzed by ImageJ and MATLAB.

### RNAi.

The replication-incompetent lentiviral vector pLKO.1 was chosen to express short hairpin RNA (shRNA) targeting BECN1. Inserted oligonucleotides were as follows: F, 5′-CCG GCT CAG GAG AGG AGC CAT TTA TTT CAA GAG AAT AAA TGG CTC CTC TCC TGA GTT TTT G-3′; R, 5′-AAT TCA AAA ACT CAG GAG AGG AGC CAT TTA TTC TCT TGA AAT AAA TGG CTC CTC TCC TAG G-3′. They were annealed and ligated into the pLKO.1 vector at AgeI and EcoRI sites, producing a final plasmid named pLKO.1-shBECN1. For stable loss-of-function experiments, lentiviral particles (pLV-shBECN1) were generated through cotransfection of pLKO.1-shBECN1 with the packaging plasmid psPAX2 and envelope plasmid pMD2.G. After viral titer determination, pLV-shBECN1 was used to infect N2a cells at an optimal MOI for 24 h, and puromycin (5 μg/ml) was subsequently added for 1 week, allowing the convenient selection of stable cell lines.

### ULK1-KO cell generation.

Two genomic RNAs (gRNAs) [gRNA386, 5′-ACC CTT GAA GAC CAC CGC GA(AGG)-3′; gRNA434, 5′-ACC CTT GAA GAC CAC CGC GA(AGG)-3′] targeting ULK1 exon 1 were cloned into the pSpCas9(BB)-2A-Puro vector and introduced into N2a cells by using X-tremeGENE HP DNA transfection reagent. At 48 h later, the populations were selected for 1 week with puromycin to sort CRISPR/Cas9 targeting clones. Knockout was tested by PCR, the T7EI assay, off-target analysis, and Western blot analysis. Primers used to detect ULK1 depletion were as follows: Ontarget386-L, 5′-GTC GAC CCT TGA AGA CCA CC-3′; Ontarget386-R, 5′-GTG TGT CAG AGC TGA GTC CC-3′; Ontarget434-L, 5′-GAA CTG GTC TGA CTT CGG GG-3′; Ontarget434-R, 5′-TCG CAA GGA CCT GAT TGG AC-3′. The Phyre2 Web portal was used for protein modeling, and the conserved domain prediction of ULK1 kinase was conducted online (https://www.ncbi.nlm.nih.gov/Structure/cdd/wrpsb.cgi).

### TEM assay.

Cells were harvested by centrifuging at 1,000 × *g* for 5 min. The cell pellets were fixed with 4% paraformaldehyde overnight at 4°C, postfixed with 1% osmium tetroxide containing 1.5% K-ferrocyanide in 0.1 M cacodylate buffer for 2 h, and dehydrated stepwise in ethanol. The dehydrated pellets were then rinsed and embedded in Epon resin for sectioning. Ultrathin sections were mounted on copper grids and stained in brass wire mesh with 2% uranyl acetate for 30 min and 0.4% lead citrate for 2 min. Images were acquired using a Zeiss LIBRA 120 transmission electron microscope operating at 120 kV.

### Statistical methods.

Two-tailed unpaired Student's *t* test and one-way analysis of variance (ANOVA) were used in this paper. All statistical tests and *P* values were analyzed by using GraphPad Prism 8.0.2 Software. P values of <0.05 (*), <0.01 (**), and <0.001 (***) were considered statistically significant, and “ns” indicates a nonsignificant difference in the figures.
